# Genetic Diversity of Imipenem-Resistant *Acinetobacter baumannii* Infections at an Intensive Care Unit

**DOI:** 10.1155/2020/3290316

**Published:** 2020-02-21

**Authors:** Amani Alnimr, Aisha Alamri, Afnan Alsultan

**Affiliations:** ^1^College of Medicine, Imam Abdulrahman Bin Faisal University, Dammam, Saudi Arabia; ^2^College of Applied Medical Sciences Imam Abdulrahman Bin Faisal University, Dammam, Saudi Arabia

## Abstract

*Introduction*. Imipenem-resistant *Acinetobacter baumannii* (IRAB) represents a major clinical threat. Dissemination in critical care areas necessitates effective action measures including genotyping tools to study the clonality of these strains and trace their origin. The main aim of this study is to assess the genetic relatedness between IRAB isolates in our institution intensive care units (ICU) which are at a particular risk of outbreaks.

**Methods:**

Nonreplicate IRAB strains were serially collected over 3 years period (January 2016–December 2018) from patients admitted to the ICU. The isolates were phenotypically identified by a matrix-assisted laser desorption/ionization time-of-flight- (MALDI-TOF-) based system (VITEK MS), and their susceptibility was tested by the phenotypic-based VITEK 2 system. Molecular fingerprinting was performed by enterobacterial repetitive intergenic consensus (ERIC-PCR) followed by hierarchal clustering. The patterns were analysed by the software of BioNumerics package version 7.6.3 (Applied Maths, Belgium).

**Results:**

A total of eighty IRAB were isolated from 31 colonization and 59 infection sites in patients admitted to the ICU. Sixty-two samples were respiratory in origin (77.5%). The generated dendrogram revealed distinct patterns for majority (95%) of the strains. Meropenem maintained activity against 43.8% of the imipenem-resistant *A. baumannii*.

**Conclusion:**

Meropenem can be a therapeutic option for imipenem-resistant *A. baumannii*.

## 1. Introduction

*Acinetobacter baumannii* is a challenging nosocomial pathogen that has adapted to the hospital environment with a particular link to critical care units [1]. A global study covering seventy-five countries in five continents identified this pathogen as the fifth problematic organism implicated in nosocomial outbreaks and healthcare-associated infections in critical care settings [2]. Infections by the multidrug-resistant (MDR) strains, defined as nonsusceptibility to an agent or more in three antimicrobial classes, as well as the extensively drug-resistant (XDR) strains of *A. baumannii* and defined as nonsusceptibility to at least one agent in all but two or fewer antimicrobial classes, are being increasingly reported in various parts of the world [3–5]. A recent systematic review that examined multiresistance in the organism over ten years has concluded that the postantibiotic era for *A. baumannii* is expected [6]. Since carbapenems are considered a last option of treatment in invasive infections by *A. baumannii*, the emergence and spread of carbapenem-resistant *Acinetobacter baumannii* (CRAB) strains is a serious clinical threat [6–8]. Outbreaks are frequently reported with CRAB in healthcare institutions and particularly in critical care areas [9]. These outbreaks can be caused by clonal dissemination or spread of genetically unrelated strains. The clonality in such a case can be examined through molecular typing tools of which several methods have been used to type the organism in different settings [10].

PCR-based genotyping schemes are rapid, inexpensive, and user-friendly tools for typing *A. baumannii* in case of outbreaks. The primers in repetitive-element PCR fingerprinting determine the repetitive DNA sequences within an organism genome. An example is enterobacterial repetitive intergenic consensus (ERIC) sequence for genotyping bacterial strains. These methods are capable of generating discriminative DNA fingerprinting tools that proved to be effective in detecting genetically related clonal lineages [11]. Studies that have highlighted the genetic relatedness of CRAB in intensive care areas of Middle-Eastern countries are limited [12–15]. This study was carried out to assess the genetic relatedness of a cohort of CRAB isolated at a major academic hospital in Eastern Saudi Arabia using ERIC-PCR-based hierarchal clustering. This can aid to ascertain nosocomial clusters and link potentially related strains of common sources among patients to better understand the population structure of *A. baumannii*.

## 2. Materials and Methods

### 2.1. Research Settings and Isolates

All nonreplicate strains of *A. baumannii* isolated from clinical specimens belonging to patients of all ages were admitted to the intensive care areas at King Fahad Hospital of the University between January 2016 and December 2018. The first specimen of a patient was used, and patients' surveillance samples upon admission or transfer were excluded. The specimens were inoculated on blood, and MacConkey agar plates (Saudi Prepared Media Laboratory Company (SPML), Saudi Arabia) incubated aerobically overnight at 35°C. Suspected colonies based on colonial morphology and catalase positive and oxidase negative reactions were further tested to confirm their identity. In addition, environmental sampling was attempted on various surfaces in the intensive care unit as previously described, which included high-touch surfaces (bedrails, over bed tables, infusion pumps, keyboards, and crash trolleys) [16]. Each environmental swab was inoculated in a 5 ml brain-heart infusion (BHI) broth (SPML, Saudi Arabia) and mixed. Following an overnight incubation at 35°C, the growth was subcultured to a MacConkey agar plate (SPML, Saudi Arabia).

### 2.2. Phenotypic Methods

Identification of *A. baumannii* was performed in the diagnostic microbiology laboratory using VITEK MS (bioMérieux, US) based on matrix-assisted laser desorption/ionization time-of-flight (MALDI-TOF) technology, while antimicrobial susceptibility testing was carried out by VITEK 2 system (bioMérieux, US). *E*-tests (AB BIODISK, Sweden) were used to measure the minimal inhibitory concentrations (MIC) for carbapenems and tigecycline as per the Clinical Laboratory Standards Institute (CLSI 2018) guidance for all antimicrobials except tigecycline where the MICs were interpreted according to the U. S. FDA breakpoints [17]. Isolates that were carbapenem-resistant (CRAB), defined as resistance to any carbapenem, were further studied. Frozen stocks of all bacterial isolates were maintained at—80°C until molecular work was conducted.

### 2.3. Genomic DNA Extraction, ERIC-PCR Fingerprinting, and Cluster Analysis

Isolates of *A. baumannii* were streaked from the frozen stocks, and a loopful of bacteria was inoculated into Luria–Bertani (LB) agar using 300 *µ*l of sterile molecular grade water and grown overnight at 37°C. Genomic DNA was extracted from overnight cultures, and the primers used for ERIC-PCR were as described by Versalovic et al. [18, 19]. Hierarchal cluster analysis was performed, and the samples were grouped into subsets based on the pairwise similarities among their ERIC profiles. Fingerprinting cluster analysis software of BioNumerics package version 7.6.3 (Applied Maths, Belgium) was used. Dendrograms were generated for the ERIC-PCR gels using Pearson correlation coefficient as a similarity measure and the unweighted pair group method (UPGMA) as a clustering algorithm with 1% optimization and 1% position tolerance. *A. baumannii* strains with a similarity exceeding 95% were considered clonally related.

## 3. Results

### 3.1. Clinical and Epidemiological Characterization of Cases of *A. baumannii*

80 nonreplicate strains of imipenem-resistant *A. baumannii* were obtained from ICU patients between 2016 and 2018. The clinical and demographic characteristics of patients with those strains are listed in Table 1. The median age was 60 years, and the median stay in the ICU prior to isolation was 15 days. All environmental cultures did not grow *A. baumannii* during the study period. Distribution of the first clinical specimen that grew CRAB from an intensive care-admitted case over 3 years is illustrated in Table 2.

### 3.2. Phenotypic Susceptibility Patterns of the *A. baumannii* Strains

Susceptibility testing results for commonly tested agents as per the CLSI for all *A. baumannii* and bacteremic strains are shown in Tables 3 and 4, respectively, with >50% cross-resistance noted for the 2 carbapenems tested.  Table 5 demonstrates the MICs of tigecycline as obtained by *E*-test (breakpoint is 2 *µ*g/ml).

### 3.3. Genotypic Characterization of *A. baumannii* Isolates by ERIC-PCR

**Figure 1:**
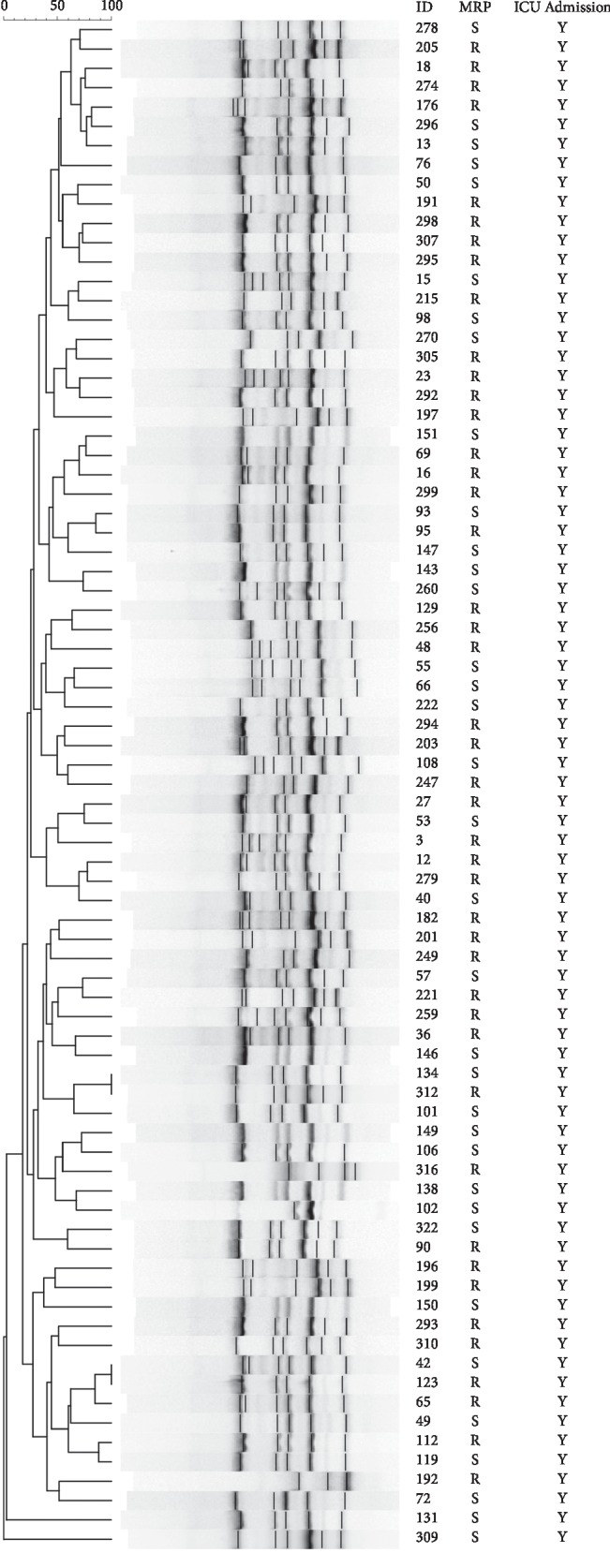
Dendrogram showing genetic diversity of 80 carbapenem-resistant *Acinetobacter baumannii* isolated from patients admitted to intensive care units between 2016 and 2018 typed by enterobacterial repetitive intergenic consensus‐polymerase chain reaction with the numbers indicating the corresponding strains followed by meropenem susceptibility status.

ERIC‐PCR fingerprinting grouped imipenem-resistant *A. baumannii* strains isolated from different specimens in patients admitted to the intensive care over 3 years period in different clusters (Figure 1). Among the 80 collected strains of *A. baumannii*, only 2 pairs of strains were found to be of similar ERIC-PCR genotypes. Strain nos. 134 and 312 originated tracheal samples of two cases admitted both in the medical ICU in May 2017 and October 2018, while isolates 42 and 123 came from tracheal samples of patients admitted in January 2017 (medical ICU) and April 2017 (surgical ICU), respectively.

## 4. Discussion

Molecular tools can contribute to the understanding of the population genetic diversity and dissemination of multidrug resistant *A. baumannii* in intensive care areas. In this study, 80 strains of imipenem-resistant *A. baumannii* were collected from critically ill patients over 3 years (2016–2018) with the majority (61.2%) being representative of true infections. The highest frequency, 60%, of the strains was obtained in 2017 which may not necessarily reflect the true incidence per 1000 patients per day as this can be influenced by the patients' days. Most of the CRAB isolates (77.5%) in our academic institution were recovered from respiratory specimens (Table 2), and all of the isolates were resistant to one or more of three or more classes of tested antibiotics fulfilling the definitions of MDR or XDR. It has been shown earlier that teaching hospitals tend to harbor more MDR organisms necessitating stringent infection control measures [20].

CRAB infections carry high mortality that was reported up to 61.8% [21, 22]. Bacteremic infections in particular have poor outcomes [22, 23]. We found a high crude mortality with blood isolates but that needs to consider that those cases of CRAB represented only a small fraction (16.3%) of the studied cohort. Limited therapeutic options play a role in worsening prognosis. In our study, most of the 13 bacteremic strains were resistant to most tested antimicrobial agents in clinical use (Table 4). Tigecycline is amongst few effective drugs in cases of nonbacteremic infections by extensively drug-resistant *A. baumannii.* No CLSI breakpoints exist for the drug in case of *A. baumannii.* In Table 5, we demonstrated that half of CRAB strains in the intensive care units of our institution have higher MICs to the drug (>2 *µ*g/ml). An important consideration is the potential overestimation of MICs of tigecycline by the methods in common use in diagnostic laboratories, namely, the *E*-test and automated systems, e.g., VITEK 2 [24]. Although aminoglycosides exhibited in vitro activity against half of the CRAB isolates, the use of this class of drugs in critically ill patients is hindered by the poor tissue penetration and renal toxicity [25].

The analysis of clonal relationships among pathogens is important to better understand the molecular epidemiology. Genotyping is more informative than antibiograms for monitoring spread of strains and early detection of outbreaks but most of the described methods are complex and labor-intensive [26]. The simplicity of ERIC-PCR makes it a useful, inexpensive tool in fingerprinting of nosocomial pathogens, and it was shown to be discriminative for *A. baumannii* with high reproducibility when coupled with the use of postprocessing data software [27]. In the present study, ERIC-PCR was performed for typing 80 strains of *A. baumannii*. The fingerprint analysis revealed the genotype diversity of CRAB isolates in the critical care units. The few isolates that were indistinguishable at 95% were chronologically and spatially unrelated making their direct dissemination less likely. This was also supported by their different meropenem susceptibility statuses and MICs to carbapenems and tigecycline. Previous published work in 2014 by Aljindan et al. examined 59 strains in our center and suggested genetic similarities amongst *A. baumannii*, but that study was conducted over a 9-month period, was hospital-wide, and included carbapenem-susceptible strains with the usage of the cutoff threshold of 90% [19]. Since the cohort presented is a homogenous collection of intensive care cases, our observations suggest that the underlying drives for the carbapenem resistance in *A. baumannii* in those cases can be related to exposure to the drugs earlier which is noted to occur more frequently in critically ill patients [20, 28]. This hypothesis needs to be tested further by correlating the antimicrobial consumption in those units and the frequency of CRAB isolation. Continuous surveillance and effective antimicrobial stewardship programs should supplement infection control measures to prevent the dissemination of CRAB clones in the critical areas [28].

The main limitations of the study are the small number of strains and the nonavailability of colistin susceptibility data which require broth microdilution testing that is not performed in routine laboratories. The visual reading of ERIC-PCR bands is subject to errors and produces doubtful results, while the use of advanced software assisted analysis, such as in this study, can solve the issue as the software has an advantage of normalizing the generated ERIC-PCR bands and hence less background noise and more accurate comparison. Future prospective, larger studies are needed to address the antimicrobial consumption in critical care areas and its effect on isolation of CRAB of diverse genotypes.

## 5. Conclusion

In this study, we have shown that ERIC-PCR fingerprint analysis of 80 strains of IRAB collected over 3-years period from ICU patients revealed distinct patterns, while no environmental reservoirs could be identified. These are likely to represent genetically unrelated strains as suggested by their phenotypic antibiogram and hierarchical clustering of ERIC-PCR profiles. The findings could be considered a representation of polyclonality in the described setting. Further work is needed to elaborate on the diversity of CRAB in hospital units with high antimicrobial consumption.

## Figures and Tables

**Table 1:** Characteristics of critically ill patients from whom the carbapenem-resistant *A. baumannii* was isolated. The frequency of isolation showed an increasing trend with age.

	No (%)
Colonization	31 (38.8)
Infection	49 (61.2)
Males	42 (52.5)
Females	38 (47.5)
Age in years	
<15	1 (1.2)
15–44	20 (25)
45–64	27 (33.8)
≥65	32 (40)
Medical ICU	53 (66.3)
Surgical ICU	27 (33.7)
30 days crude mortality^*∗*^	15 (18.8)

^*∗*^30 days crude mortality from 13 cases of blood stream infections by carbapenem-resistant *A. baumannii* was 61.5%.

**Table 2:** Distribution of clinical isolates according to sample type and year in the intensive care units over the study period.

Year	2016	2017	2018	Total
Respiratory	4	37	21	62
Blood	2	9	2	13
Skin/soft tissues	1	1	1	3
Others	1	1	0	2
Total	8	48	24	80

**Table 3:** Susceptibility profiles of 80 strains of carbapenem-resistant *A. baumannii* isolated from intensive care cases.

Antimicrobial	Susceptibility rates %
Ceftazidime	0
Cefepime	0
Piperacillin-tazobactam	0
Imipenem	0
Meropenem	43.8
Gentamicin	53.8
Amikacin	53.8
Ciprofloxacin	0
Levofloxacin	5
Trimethoprim-sulfamethoxazole	8.8

**Table 4:** Susceptibility profiles of 13 strains of carbapenem-resistant *A. baumannii* isolated from blood stream infections in the intensive care. ^*∗*^MIC ranges for meropenem 1–256 *µ*g/ml.

Antimicrobial	Susceptibility rates %
Ceftazidime	0
Cefepime	0
Piperacillin-tazobactam	0
Imipenem	0
Meropenem	46.2^*∗*^
Gentamicin	46.2
Amikacin	46.2
Ciprofloxacin	0
Levofloxacin	7.7
Trimethoprim-sulfamethoxazole	23.1

**Table 5:** Minimal inhibitory concentrations of tigecycline against 80 strains of carbapenem-resistant *Acinetobacter baumannii (*MIC50 = 2 *µ*g/ml, MIC90 = 4 *µ*g/ml). Tigecycline susceptible strains represented 53.8% of the complete cohort.

MIC (*µ*g/ml)	No. of isolates (%)	Cumulative no. of isolates (%)
1	12 (15)	12 (15)
2	31 (38.8)	43 (53.8)
3	26 (32.5)	69 (86.3)
4	4 (5)	73 (91.3)
6	1 (1.3)	74 (92.5)
8	2 (2.5)	76 (95)
16	3 (3.8)	79 (98.8)
32	1 (1.3)	80 (100)

## Data Availability

The data used to support the findings of this study are available from the corresponding author upon request.
